# The Burden of Tick-Borne Encephalitis in Disability-Adjusted Life Years (DALYs) for Slovenia

**DOI:** 10.1371/journal.pone.0144988

**Published:** 2015-12-16

**Authors:** Renata Šmit, Maarten J. Postma

**Affiliations:** 1 Department of Pharmacy, Unit of PharmacoEpidemiology & PharmacoEconomics (PE2), University of Groningen, Groningen, Netherlands; 2 Institute for Science in Healthy Aging & healthcaRE (SHARE), University Medical Center Groningen (UMCG), University of Groningen, Groningen, Netherlands; 3 Department of Epidemiology, UMCG, University of Groningen, Groningen, Netherlands; University of Minnesota, UNITED STATES

## Abstract

**Background:**

Tick-borne encephalitis (TBE) presents an increasing burden in many parts of Europe, Asian Russia, Siberia, Asian former USSR and Far East. Incidence can be considered as one way to express the burden. A more comprehensive measure concerns disability-adjusted life years (DALYs), better characterizing the full burden of TBE. TBE burden in DALYs has not yet been estimated, nor has it been specified by the Global Burden of Disease (GBD) studies.

**Objective:**

The purpose of the present study is to estimate the burden of TBE in Slovenia, expressed in DALYs, both from the population and individual perspectives. We discuss the impact of TBE burden on public health and potential strategies to reduce this burden in Slovenia.

**Methods:**

The burden of TBE is estimated by using the updated DALYs' methodology first introduced in the GBD project. The DALYs᾽ calculations are based on the health outcomes of the natural course of the disease being modelled. Corrections for under-reporting and under-ascertainment are applied. The impact of uncertainty in parameters in the model was assessed using sensitivity analyses.

**Results:**

From the population perspective, total DALYs amount to 3,450 (167.8 per 100,000 population), while from the individual perspective they amount to 3.1 per case in 2011. Notably, the consequences of TBE present a larger burden than TBE itself.

**Conclusions:**

TBE presents a relatively high burden expressed in DALYs compared with estimates for other infectious diseases from the GBD 2010 study for Slovenia. Raising awareness and increasing vaccination coverage are needed to reduce TBE and its consequences.

## Introduction

Tick-borne encephalitis (TBE) presents an increasing public health concern in many parts of Europe and in some Asian countries [1]. TBE is an infectious disease of the central nervous system (CNS) which may lead to long term or permanent neurological sequelae or even death [2–6]. Three genetically closely related TBE virus (TBEV) subtypes (the European, Far-Eastern and Siberian) cause TBE [1,7]. These three TBE virus subtypes are usually transmitted by the infected tick genus *Ixodes* [1]. The virus can also be transmitted through unpasteurized dairy products [8]. All three subtypes are associated with varying courses of the disease and degrees of severity [7]. The European subtype of the virus, which is prevalent in most of Europe (including Slovenia), usually causes disease that follows a biphasic course [7]. This course presents a short-lasting acute disease of the first stage with flu-like symptoms, starting about two weeks after the tick bite, followed by a few symptom-free days with subsequent acute disease of the second stage in the CNS, mostly concerning meningitis, meningoencephalitis and meningoencephalomyelitis [9]. With increasing age, severity of the disease increases [9,10] and mortality rates due to TBE in adults increasing up to 2% [7]. The Far Eastern subtype of the virus is mainly found in Eastern Russia, China, and Japan and the Siberian is found mostly in the Urals, Siberia and Eastern Russian provinces [7]. Both the Far Eastern and Siberian subtypes of the virus cause a monophasic course of the disease [7], characterized by CNS involvement [7]. Disease with the Far Eastern subtype of the virus is usually severe, is frequently associated with encephalitic symptoms and lacks the tendency to develop chronic disease [7]. Mortality rate due to the Far Eastern subtype is between 5 and 35% [7]. Disease from the Siberian subtype of the virus is less severe, but associated with diverse neurological and/or neuropsychiatric symptoms and a tendency to develop chronic or extremely prolonged disease [7]. Mortality rate of the Siberian subtype is between 1 and 3% [7]. Finally, the disease characteristics also depend on the tropism of the TBEV for different regions of the CNS and on the individual immunity of patients [9–11].

TBE is endemic in regions from Alsace-Lorraine and Scandinavia to the North-eastern parts of China and Northern Japan [1,12–19]. In many parts of Europe, Russia, Siberia and the Far East, the incidence is increasing and new foci have appeared due to increasing mobility, changes in lifestyle, human leisure activities, agricultural practices and effects of climate changes on vectors and reservoir hosts [20–24]. In spite of increasing awareness and knowledge of the disease, the incidence of TBE is underestimated [21], mainly due to insufficient routine diagnostics and surveillance [9]. Therefore the full burden of TBE, using corrections for this underestimation, still has to be assessed. Vaccination with safe, efficacious and well tolerated vaccines is the most effective way of preventing TBE [25–28] and reducing its burden. In Slovenia, TBE has been a relevant disease from the public-health point of view since 1953 [29]. Slovenia is one of the countries where the European subtype of the virus is prevalent, with a high incidence of TBE and very low vaccination coverage [30,31] despite favourable cost-effectiveness [32]. Though the cost-effectiveness of vaccination against TBE in Slovenia was evaluated [32] the exact full burden of TBE is also still unknown in this country and needs to be estimated.

The burden of TBE is mostly expressed by incidence only [9,30,33]. The burden of the disease can be better evaluated if information on the incidence/prevalence, mortality and sequelae are all combined into a composite measure which can be found in the disability-adjusted life years (DALYs) [34]. DALYs reflect lost years of healthy life, as defined by the World Health Organization (WHO) [35]. DALYs, as one criteria, can be used for effective planning and prioritizing of scarce and therefore limited public health resources [34]. The burden of TBE, expressed in DALYs, has not yet been assessed nor has it been specified in the Global Burden of Disease (GBD) studies [36].

The purpose of the present study is to estimate the burden of TBE in Slovenia, expressed in DALYs from both the population and individual perspectives. Furthermore, we discuss strategies to reduce this public health burden in Slovenia. Data used reflect 2011 as the most recent year with data availability. Additionally, we consider the trend in DALYs to analyse how reflective 2011 is for other recent years. Our study can help to guide health policy and action locally as well as provide suggestions globally to mitigate the burden of TBE.

## Methods

### Brief overview of the DALYsʼ methodology

The DALYsʼ methodology was first introduced by Murray and co-workers in the Global Burden of Disease (GBD) project [37] using the following equation:

DALYs=YLLs+YLDs (1)

where YLLs are the number of life years lost due to premature death and YLDs are the number of life years lost due to disability, weighted with a factor between 0 (perfect health) and 1 (death) reflecting the severity of the disability. According to the GBD-terminology and the International Classification of Functioning, Disability and Health, [38] the term disability refers to any short-term or long-term health loss in terms of functional capacity such as mobility, self-care, participation in normal activities, pain and discomfort, anxiety and depression and cognition.

In the original GBD study in 1990 and further WHO updates [39–47], 3% and 0% discount rates and both presence and absence of age weights in the DALYs’ calculations were used. Discounting means that the value of health is weighted less in the future than in the present due to time preference related to growth in life expectancy [48]. Age weights give less weight to years of healthy life lost at young ages and older ages, with productivity as the main rationale [48]. For the GBD 1990 study, the reference standard life table has a life expectancy at birth of 82.5 years for females and 80.0 years for males [48].

Since the GBD 1990 study was published, there have been intense debates on the key methodological choices used for the DALYs’ calculation, especially regarding the discounting, age weights, disability weights, the years lost per death and the incidence estimates for the YLDs calculation [48–54]. As one outcome of these debates, the new GBD 2010 study, done by Institute of Health Metrics and Evaluation, used a simplified calculation of DALYs without discounting and age weights, and the YLDs are calculated from prevalence estimates rather than incidence estimates [48,55–58]. The GBD 2010 study also involves some modifications of disability weights for the DALYsʼ calculus and uses a new reference standard life table for the YLLs calculation. The new GBD 2010 reference standard life table has a life expectancy at birth of 86 years for both males and females and expresses an aspiration for high and healthy life expectancy [55].

The GBD studies estimate the global burden of diseases, including communicable, maternal, neonatal, nutritional, non-communicable diseases and injuries [48]. In 2008, the European Centre for Disease Prevention and Control urged to develop a new methodology to estimate the burden for communicable diseases in European Member States and EEA/EFTA countries by using a pathogen-based approach, taking into account subsequent sequelae and complications associated with infection [59,60]. No age weights or discounting are considered [59]. Adjustment to correct for under-ascertainment and under-reporting is applied [59,61]. Under-ascertainment refers to cases who do not seek medical care due to mild or absence of symptoms, or who have knowledge that the disease is self-limiting [61,62]. Due to under-ascertainment, cases will not enter the notification or surveillance system [61,62]. Under-reporting refers to cases who seek medical care but are not captured by the surveillance or notification systems because the infection/pathogen is not diagnosed or is misdiagnosed, misclassified or miscounted [61,62]. Further development of this methodology is in progress [60]. Using this methodology to estimate the full burdens of different communicable diseases enables comparisons among these diseases within and between countries to help guide public health policy and action in Europe [59]. In the present study, the updated methodology [60,61,62] will be applied for one individual country.

### The DALYs methodology to calculate the burden of TBE

On the basis of the natural course of the disease, the following health outcomes of a model were included for the calculation of DALYs [32]: death due to TBE, the acute disease of the second stage with signs of meningitis, meningoencephalitis and/or meningoencephalomyelitis and mild, moderate and severe neurological sequelae. Mild neurological sequelae (emotional liability, tiredness and intermittent headache) can be assumed not to have a significant impact on patientsʼ daily activities, social and working capacity. Moderate neurological sequelae (ataxia of gait, paresis of extremities, cognitive disorders, pronounced dementia or severe deafness) will however affect patients’ daily activities, social and working capacity. In patients with severe neurological sequelae, social life and working capacity can be seriously affected and in a few cases patients need institutional care. In the present study, these neurological sequelae are considered as permanent neurological sequelae.

**Fig 1:**
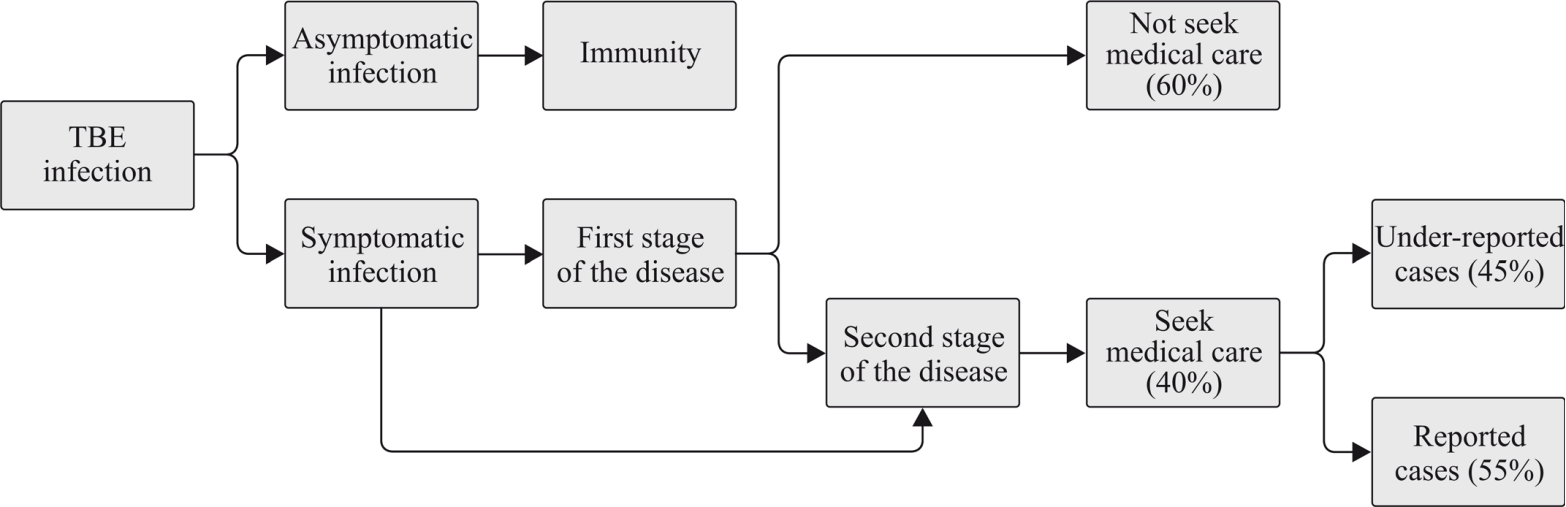
Schematic presentation of disease progression, used to estimate the factors β_r,_ β_a_ and consequently γ; with γ (4.5) to correct under-estimation of the reported number of cases with the acute disease of the second stage (n_ra_) resulting from the product of a factor β_a_ (100/40 = 2.5) to correct for under-ascertainment and a factor β_r_ (100/55 = 1.8) to correct for under-reporting.

Data in Table 1 were used to calculate the burden for TBE in a straightforward disease burden model programmed in Microsoft Excel. The reported age-dependent number of cases with acute disease of the second stage (n_ra_) was obtained from the National Institute of Public Health (NIJZ) data [63] and was used as the initial model input. These numbers of cases were corrected for under-estimation by a factor γ. This factor γ is the product of a factor β_a_ to correct for under-ascertainment and a factor β_r_ to correct for under-reporting. Both correction factors β_a_ and β_r_ are calculated based on the schematic presentation of disease progression presented in Fig 1. [32]. In the present study the factor β_a_ was calculated on the basis of targeted specific information. Firstly, the natural course of TBE takes into account that around 90% of symptomatic infections develops acute disease of the first stage with symptoms similar to flu and about one third develops acute disease of the second stage with CNS involvement and the remaining 10% of infections involves more serious disease of the CNS [33,64]. Secondly, all reported cases were assumed to concern patients with acute disease of the second stage with CNS involvement in Slovenia [63].

**Table 1:** Model input parameters for calculating DALYs of TBE: the number of reported cases with acute disease of the second stage for 2011, duration of the acute disease of the second stage, disability weights for the acute disease of the second stage, probabilities of permanent neurological sequelae, disability weights for permanent neurological sequelae and probabilities of death from TBE, inclusive references.

Health Outcomes	Base-case	Range	Reference (base-case)
Acute disease of the second stage			
Correction factor for underestimation (γ)	4.5 ^a^	3.5–5.5 ^b^	
Number of all acute cases, by age groups			
0–4	5		[63]
5–14	17		[63]
15–24	20		[63]
25–34	19		[63]
35–44	34		[63]
45–54	38		[63]
55–64	65		[63]
65–74	41		[63]
75+	8		[63]
Duration of hospitalization, by age groups ^c^			
0–14	0.0164 years (6 days)	0.0082 years (3 days)-0.0274 years (10 days) ^d^	[70]
15–64	0.0247 years (9 days)	0.0082 years (3 days)-0.1671 years (61 days) ^d^	[71]
65–74	0.0274 years (10 days)	0.0082 years (3 days)-0.1205 (44 days) ^d^	[71]
75+	0.0274 years (10 days)	0.0082 years (3 days)-0.1205 (44 days) ^d^	[71]
Disability weights, by age groups ^e^			
0–14	0.616 per life year lived with disability		[68]
15–74	0.613 per life year lived with disability		[68]
75+	0.613 per life year lived with disability		[68]
**Permanent neurological sequelae**			
Probability of mild sequelae, by age groups			
<15	0%		[29]
>15	10%	10–15.4% ^f^	[65]
Probability of moderate sequelae, by age groups			
<5	0%		[29]
5–14	1.50%	0–1.5% ^g^	[29]
>15	46%	22–46% ^f^	[65]
Probability of severe sequelae, by age groups			
<5	0%		[29]
5–14	0.80%	0–4.1% ^g^	[29]
>15	2%	2–8.6% ^f^	[65]
Duration	Remaining life expectancy in years		
Disability weights, all ages ^h^			
Mild sequelae	0.023 per life year lived with disability	0–0.1 per life year lived with disability	
Moderate sequelae	0.160 per life year lived with disability	0.1–0.25 per life year lived with disability	
Severe sequelae	0.629 per life year lived with disability	0.5–0.7 per life year lived with disability	
**Death due to TBE**			
Correction factor for underreporting (β_d_)	2^i^	0–4 ^j^	
Probability of death from TBE	0.4% ^k^		
Duration	Remaining life expectancy in years		
Disability weights	1 per life year lived with disability		[36]

a The calculated factor γ (4.5) is a product of factor βa (100/40 = 2.5) and factor βr (100/55 = 1.8). The factor βa was calculated on the basis of: (i) the natural course of TBE [33,64] and (ii) all reported cases in Slovenia (patients with acute disease of the second stage with CNS involvement). The factor βr for TBE in Slovenia is not available, therefore βr (100/55 = 1.8) is calculated taking into account that 55% TBE cases are reported in Slovenia.

b The calculated average coefficient of variation (23%) of reported cases during the period 2002 to 2011 [63] was used for calculating the maximum and the minimum values of the factor γ. As it is stated that 97% of all reported cases are hospitalized in 2011 [63], a simplified assumption that all reported cases are hospitalized is used.

c The age groups available for duration of hospitalization at 15 to 60 years and >60 years [71] is adapted into a set of age groups of 15–24, 25–34, 35–44, 45–54, 55–64, 65–74 and 75+ years based on official sources of the NIJZ [63].

d The maximum and the minimum values for duration of hospitalization by all age group, were taken from published studies in Slovenia [70,71].

e Disability weights for acute disease of the second stage are not available, therefore the disability weights of Japanese encephalitis from GBD 2004 were updated and subsequently taken into account [68].

f The maximum and minimum values of probabilities for mild, moderate and severe permanent neurological sequelae for those aged 15 years and over were taken from the highest and lowest values from both studies in Slovenia [65] and Lithuania [4].

g The maximum and the minimum values of probabilities for mild, moderate and severe permanent neurological sequelae for under the age of 15 years were taken from both studies in Slovenia [72,73].

h Disability weights for mild, moderate and severe permanent neurological sequelae due to TBE are not available, therefore the disability weights for each of these sequelae as well as their maximum and minimum values were calculated as 1 minus available quality weights. The quality weights for mild, moderate and severe permanent neurological sequelae and their maximum and the minimum values were derived from published data [69].

i The factor βd (100/50 = 2) was calculated taking into account that 50% TBE deaths are reported [66].

j The maximum and minimum values for factor βd is calculated as ± 2 times the base case value.

k The probability of death due to TBE was calculated from the average reported deaths due to TBE during the period 2002 to 2011 (1) [63] divided by all reported TBE cases from 2011 (247) [63].

Specifically, factor β_r_ was calculated taking into account that 55% of TBE cases are reported; i.e., 45% of TBE cases are assumed misdiagnosed, misclassified or miscounted. The age-dependent estimated numbers of cases with acute disease of the second stage (n_ea_) were calculated as follows:

$$n_{ea}\;=\;n_{ra}\;\times \;\gamma \;=\;n_{ra}\;\times (\beta_{a}\;\times \;\beta_{r})$$ (2)

The estimated number of cases for mild (n_emild_), moderate (n_emod_), and severe (n_esev_) permanent neurological sequelae and deaths due to TBE (n_ed_) were calculated, using the annual transition probabilities p_mild_, p_mod_, p_sev_ and p_d_ of moving from acute disease of the second stage to health outcomes (mild, moderate, severe permanent neurological sequelae and deaths due to TBE, respectively). The age-dependent annual transition probabilities for mild, moderate, and severe permanent neurological sequelae were obtained from national studies [29,65]. The age-dependent annual probabilities of deaths due to TBE (p_d_) were calculated from the NIJZ data [63] and were corrected for under-reporting due to under-diagnosis by a factor β_d_. The factor β_d_ for TBE was calculated based on a published study [66] So, the age-dependent estimated numbers of cases for mild, moderate, severe permanent neurological sequelae and deaths due to TBE (n_ed_) were calculated as follows:

$$n_{emild}\;=\;p_{mild}\;\times \;n_{ea}$$ (3)

$$n_{emod}\;=\;p_{mod}\;\times \;n_{ea}$$ (4)

$$n_{esev}\;=\;p_{sev}\;\times \;n_{ea}$$ (5)

$$n_{ed}\;=\;p_{d}\;\times \;n_{ea}\;\times \;\beta_{d}$$ (6)

These estimated numbers of cases for the various health outcomes were subsequently incorporated in the DALYs calculation for TBE. YLLs were calculated by multiplying the estimated age specific numbers of deaths due to TBE (n_ed_) with the remaining life expectancy (e) at that age. For life expectancy, the latest GBD 2010 reference standard life table [58] was used.

$$YLLs=n_{ed}\;\times \;e$$ (7)

The YLDs_acute_ for the acute disease of the second stage were calculated by multiplying the estimated number of cases with acute disease of the second stage (n_ea_) with the appropriate disability weights (Dw_a_) and duration of hospitalization (l_a_).

$${YLDs}_{acute}\;=\;n_{ea}\;\times \;{Dw}_{a}\;\times \;l_{a}$$ (8)

The YLDs_seq_ for mild, moderate and severe permanent neurological sequelae were calculated by multiplying the estimated number of cases for mild (n_emild_), moderate (n_emod_) and, severe (n_esev_) permanent neurological sequelae with its disability weights (Dw_mild_, Dw_mod_ and Dw_sev_) and remaining life expectancies (e_mild_, e_mod_ and e_sev_).

$${YLDs}_{seq}\;=\;n_{emild}\;\times \;{Dw}_{mild}\;\times \;e_{mild}+\;n_{emod}\times \;{Dw}_{mod}\;\times \;e_{mod}\;+\;n_{esev}\;\times \;{Dw}_{sev}\;\times \;e_{sev}$$ (9)

The YLDs for all health outcomes (acute disease of the second stage, mild, moderate and severe permanent neurological sequelae) were aggregated to get the total YLDs.

$$YLDs\;=\;{YLDs}_{acute}\;+\;{YLDs}_{seq}$$ (10)

The full burden for TBE is the sum of YLLs and YLDs as follows:

$$DALYs\;=\;n_{ed}\times e\;+\;n_{ea}\;\times \;{Dw}_{a}\;\times \;l_{a}\;+\;n_{emild}\;\times \;{Dw}_{mild}\;\times \;e_{mild}+\;n_{emod}\times \;{Dw}_{mod}\;\times \;e_{mod}\;+\;n_{esev}\;\times \;{Dw}_{sev}\;\times \;e_{sev}$$ (11)

The burden of the disease for one year is presented from the population (total DALYs and DALYs per 100,000 populations) and the individual perspectives (DALYs per case) [62].

Neither discounting nor age weighting was applied in the DALYs calculus.

### Data

The number of TBE cases for 2011 for age groups from 0–4 years, 5–14 years, 15–24 years, 25–34 years, 35–44 years, 45–54 years 55–64 years, 65–74 years and 75+ years originated from official sources of the NIJZ [63]. In the present study, a maximum age of 105 years was taken, corresponding to the maximum age of 105 years from the GBD 2010 new standard life table. In the present study, the ages of onset for the acute disease of the second stage and mild, moderate and severe permanent neurological sequelae were assumed at the mid-points of each age group.

We used the new GBD 2010 reference standard life table for the calculation of YLLs, with a life expectancy at birth of 86 years for males and females [58]. An incidence-based approach was used to estimate YLDs. In the absence of discounting and age weights, this approach converges to incidence multiplied by estimated duration times with disability weighting for sequelae [67]. In a prevalence-based approach without discounting and age weights, YLDs would follow from prevalence of sequelae times disability weights [67]. As prevalence is approximately incidence times duration, our incidence-based YLDs across all ages would approximately equal the prevalence-based YLDs, in particular in the absence of discount and age weighting [67]. Notably, when discounting and age weights are applied, the prevalence-based YLDs for all ages may be quite different from the incidence-based YLDs [67].

As no disability weights for the acute disease of the second stage of TBE exist, age-dependent disability weights were derived from the GBD 2004 updated study for Japanese encephalitis [68]. Also, no disability weights for the various levels of TBE-related neurological sequelae severities exist, and such disability weights for the mild and moderate and severe permanent neurological sequelae were derived as 1 minus the respective quality weights for each of these sequelae. The quality weights for mild, moderate and severe permanent neurological sequelae were derived from a published study [69]. As mentioned, all base-case parameter values are listed in Table 1.

In sensitivity analyses, we tested how the results change with changes in the input parameters’ values. One-way and probabilistic sensitivity analyses (PSA) were conducted. With one-way sensitivity analyses one parameter was changed while other parameters were kept constant at the base case values. The maximum and minimum values of each parameter were defined by the range from Table 1. The results of one-way sensitivity analyses were represented as a tornado diagram for the population perspective. PSA was conducted in the form of simulations using @Risk^®^ (Palisade Corporation) and 1000 repetitions. Uniform distributions were used between maximum and minimum values as defined by the ranges from Table 1. Results of the PSA were presented using 95%-uncertainty cut-offs.

## Results

### Base-case analyses

Table 2 shows that from the population perspective total DALYs amount to 3,450 or 167.8 per 100,000 population, while from the individual perspective they amount to 3.1 per case. The disease burden is dominated by permanent neurological sequelae (93.9%), followed by the burden of premature death due to TBE (5.6%), while the burden due to the acute disease of the second stage reflects the smallest proportion (0.5%). Within the total permanent neurological sequelae, the burden of moderate sequelae presents the biggest proportion (77.6%) followed by the burden of severe sequelae (14.0%) and the burden of mild sequelae (2.4%).

**Table 2:** Calculated DALYs due to TBE for one year from the population perspective (YLLs, YLDs, DALYs and DALYs per 100,000 population) and individual perspective (DALYs per case).

Health outcomes	Cases	YLLs	YLDs	DALYs	DALYs per case	DALYs per 100,000 population
**Acute disease of the second stage**	1,112	0	17	17	0.015	0.8
**Permanent neurological sequelae**						
Mild sequelae	101	0	83	83	0.075	4.0
Moderate sequelae	467	0	2,672	2,672	2.403	129.9
Sever sequelae	21	0	484	484	0.435	23.5
**Total permanent neurological sequelae**			3,239	3,239	2.913	157.5
**Death due to TBE**	9	194	0	194	0.174	9.4
**All health outcomes**		**194**	**3,256**	**3,450**	**3.1**	**167.8**

Cases = estimated cases. Results in the Table 2 are presented in both disaggregated form (YLLs, YLDs) and aggregated form (DALYs) for one year and are in line with the methodology protocol for calculating the burden of communicable diseases in EU and EEA/EFTA [62]. YLLs = the number of life years lost due to premature death due to TBE at an age of 67 years [30]. YLDs = the number of life years lost due to disability. DALYs = disability-adjusted life years. DALYs are calculated as the sum of YLLs and YLDs. DALYs per case are calculated as DALYs divided by the number of estimated cases of acute disease of the second stage. DALYs per 100,000 populations are calculated as DALYs divided by the Slovene population of 2,050,189 people [74] and then multiply by 100,000.

**Fig 2:**
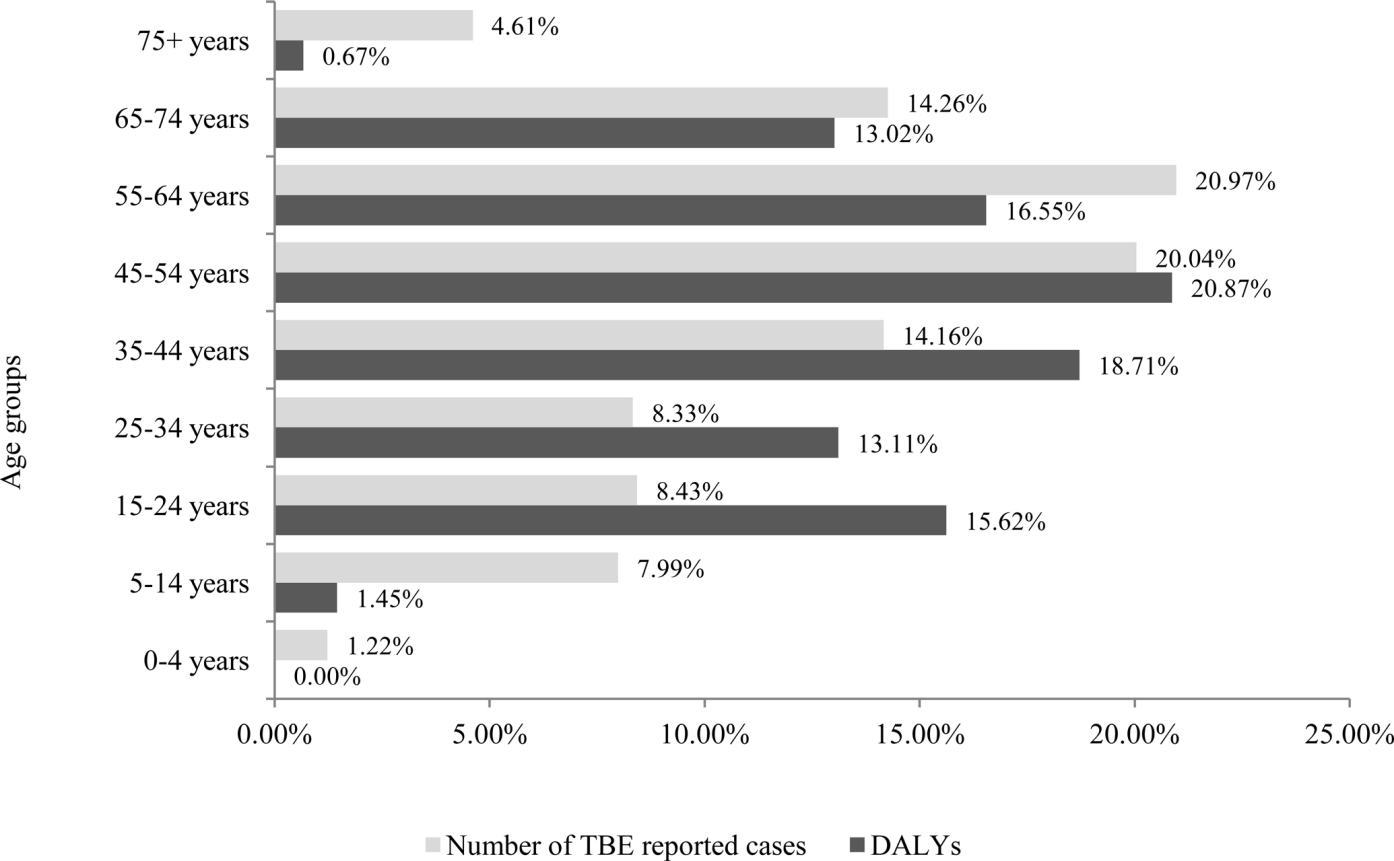
Comparison of relative TBE burden expressed in total DALYs from the population perspective with the relative TBE burden expressed by the number of reported cases by age groups. DALYs and reported cases are averages over 2004–2011.

Fig 2 shows that, from the population perspective, the burden of TBE expressed in DALYs in the age group from 15 to 54 years amounts to 68% of the total burden. The burden in the working age population from 15 to 64 years amounts to 85%. Within working age populations, the burden in the prime-aged workers group from 25 to 54 years of age amounts to 53%, followed by the burden in the youth workers group from 15–24 years of age and older workers group from 55 to 64 years (16–17%).

The burden expressed as the number of reported cases in the age group from 15 to 54 years amounts to 51% of the total burden and to 72% in the working age population aged 15 to 64 years. The burden in the prime-aged workers group amounts to 43%, followed by the burden in the older workers group (21%), while the burden in youth workers group amounts to 8%.

**Fig 3:**
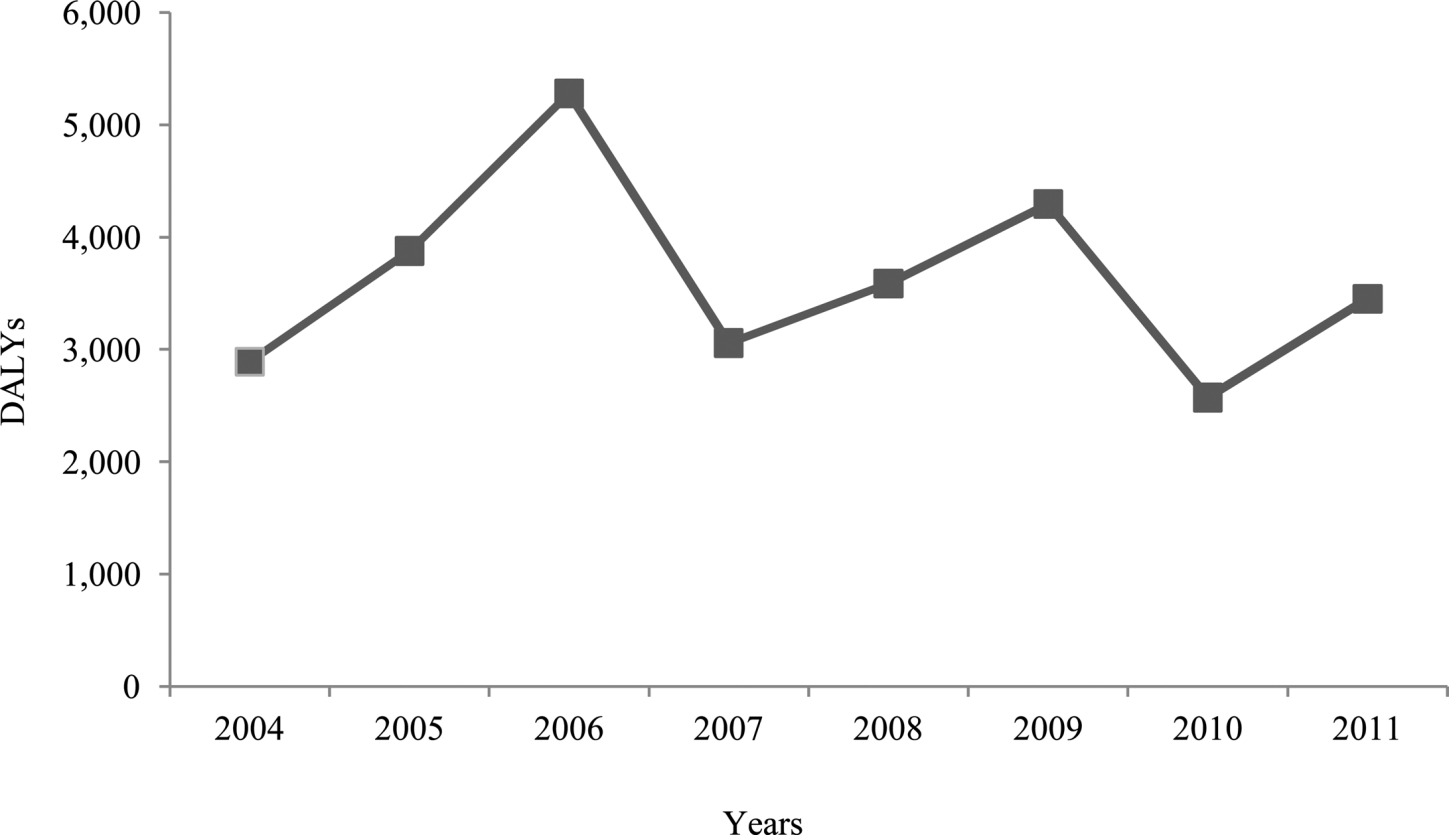
Trend analysis of TBE burden expressed in total DALYs in years 2004 to 2011 from the population perspective.

Fig 3 demonstrates annual data of total DALYs over time, with the peak of 5,279 DALYs in 2006 and the lowest value of 2,572 DALYs in 2010. Long-term burden average between 2004 and 2011 amounts to 3,626 DALYs while a short-term average between 2009 and 2011 amounts to 3,439 DALYs. Our 2011 estimate can therefore be considered as representative for previous years, despite a slight overall decline during the years.

### Sensitivity analyses

**Fig 4:**
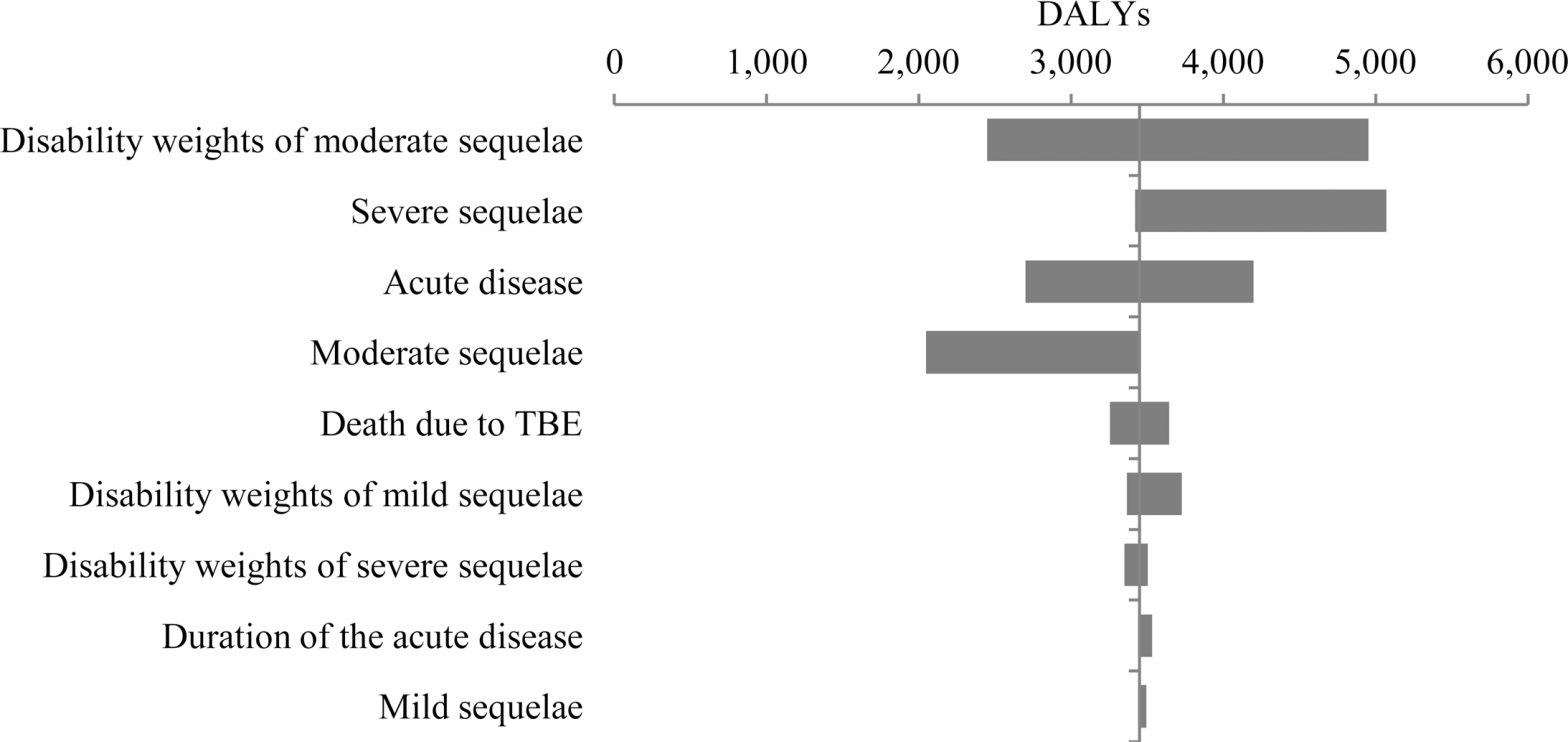
Tornado diagram of the one-way sensitivity analyses of TBE burden expressed in total DALYs from the population perspective.

With one-way sensitivity analyses, it was tested which parameters have the greatest influence on the modelʼs results. From Fig 4, the ordering of parameters according to their influence on DALYs can be seen. The disability weights of moderate permanent neurological sequelae have the greatest influence on estimated DALYs.

**Fig 5:**
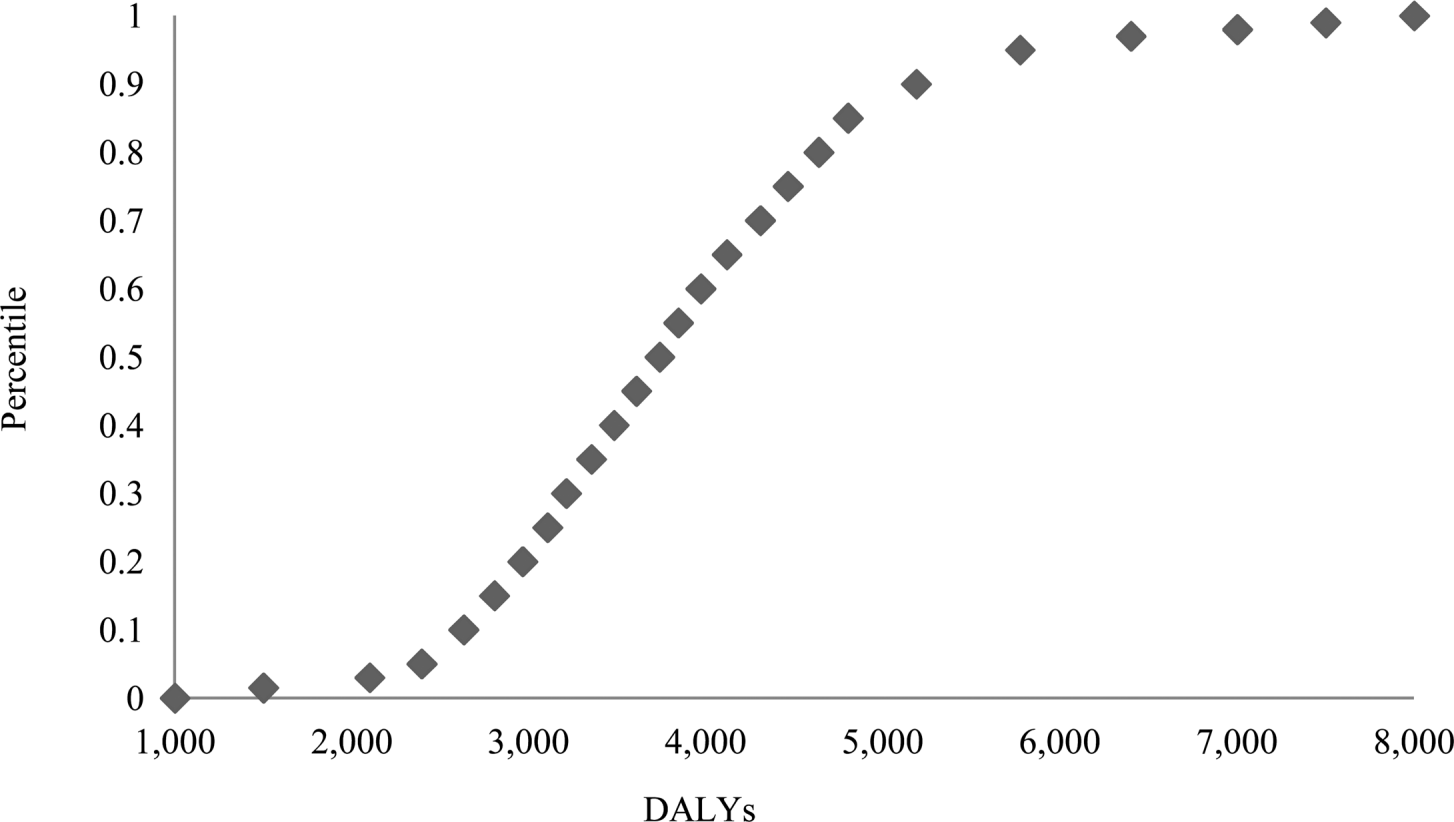
Cumulative ascending curve of the probabilistic sensitivity analysis of TBE burden expressed in total DALYs from the population perspective, reflecting the likelihood (y-axis) of being below a specified total DALYs impact (x-axis).

Results of the probabilistic sensitivity analyses presented in Fig 5 show a 95% uncertainty interval from 2,394 to 5,774 DALYs. It shows that, for example, with 0.95 likelihood total DALYs are higher than 2,394, whereas DALYs are higher than 2,631 with 0.9 likelihood.

## Discussion

DALYs as a composite measure enable the combination of information on incidence, mortality and sequelae associated with TBE infection. Therefore, DALYs provide unique insight into the disease burden [34]. From the population perspective, total DALYs amount to 3,450 (167.8 per 100,000 population) in 2011 in Slovenia, while from the individual perspective they amount to 3.1 per case for that same year. The majority of the estimated burden is centered in adults with potential major impact on productivity losses. 1.5% of TBE burden in the age group from 5–14 years seems low compared to the burden in adults. However, as these are children and disease in children is often considered as of even higher subjective burden than in adults, a greater relative burden of disease may well be conceived than reflected just by the bare percentages. Also, the present study emphasizes the impact of neurological sequelae. These sequelae have significant impact on patients᾽ quality of life, social and working activities. Cognitive impairments are perhaps one of the most relevant sequelae of TBE and potentially are crucial in the work environment.

In the present study, TBE country-specific data were used for the DALYs calculation. The study estimates are based on the best evidence the authors could find, however, some data and information were not available for Slovenia, such as disability weights for TBE, both correction factors β_d_ (under-reporting of deaths due to TBE) and β_r_ (under-reporting of cases of the acute disease of the second stage). In Slovenia, only cases with the acute disease of the second stage with CNS [75] and death due to TBE [63] are routinely reported, DALYs have previously not been used to measure the burden of TBE and application of correction factors for TBE as done in the present study reflects an innovative approach in DALYs’ calculations. The disease burden model developed in the present study could be used as a representative model for TBE DALYs’ calculations for other countries. Cases with asymptomatic infection and symptomatic cases including acute disease of the first stage are not captured by notification or surveillance systems in Slovenia [75] nor by our correction factors, causing potential under-estimation of our DALYs’ estimates.

Disability weights of mild and severe permanent neurological sequelae, as addressed in the one-way sensitivity analyses, appeared to have no crucial impact on DALYs. Additionally, correction for under-reporting of deaths due to TBE shows only a slight impact on YLLs in the full disease burden. It is estimated that at the European level (Albania, Austria, Croatia, the Czech Republic, Denmark, Estonia, Finland, France, Germany, Hungary, Italy, Latvia, Lithuania, Poland, Russian Federation, Slovak Republic, Slovenia, Sweden, Switzerland) only 30–40% of TBE cases are reported [76], meaning that 60–70% of TBE cases are misdiagnosed, misclassified and miscounted. These figures are derived from varying systems of TBE surveillance and notification and varying diagnostic procedures among these countries in the investigated time period up to 2000 [7]. As TBE has been mandatory notifiable since 1977 in Slovenia [7] and awareness about TBE might increased in the last years, we decided to use a conservative estimate of 55% of TBE cases being reported in Slovenia. Taking into account the proportion of 30–40% of TBE reported cases [76], our total DALYs estimation would obviously still reflect an under-estimate.

Trend analysis of the TBE burden may be interpreted as showing a slight decrease in DALYs for the time period between 2004 and 2011, likely mainly due to increasing awareness of the disease among the general population. Despite increasing awareness during the last years, vaccination coverage of 13% in terms of persons receiving one or two doses of TBE vaccine was low in 2009 [77]. Notably, vaccination seems far too low in Slovenia for efficient prevention and control of TBE [31]. Still, the trend analysis shows that the estimated burden of 3,450 DALYs for 2011 is likely representative for the national disease burden.

In a report of the Dutch National Institute of Public Health & the Environment, burdens are presented as measured in DALYs, incidence and mortality for seven selected infectious diseases (influenza, measles, HIV, campylobacteriosis, infection with enterohaemorrhagic *Escherichia coli*, salmonellosis and tuberculosis) and compared between some European countries [34]. It was concluded that the relative burden of these diseases expressed in DALYs is different compared to the relative burden expressed just by incidence or mortality data [34]. In a related study [78] explicitly recommend to calculate DALYs also for other infectious diseases in Europe to prioritize interventions.

DALYs have previously not been used to measure the burden of TBE. TBE burden, expressed in DALYs is not included in GBD studies, and results of the present study give novel information in that respect. Comparison of the TBE burden expressed in DALYs in the present study to DALYs for other infectious diseases from the GBD 2010 study for Slovenia [79] is possible. However, this comparison should be considered with caution as no GBD disability weights for TBE exist. In the GBD 2010 study for Slovenia [79], the cluster for communicable, maternal, neonatal and nutritional disorders presents 25,501 DALYs per year. Within this group [79], lower respiratory infections present the highest burden with 7,545 DALYs amounting to 29.6%, followed by diarrheal diseases with 1,225 DALYs (4.8%), otitis media with 954 DALYs (3.7%), upper respiratory infections with 951 DALYs (3.7%), tuberculosis with 776 DALYs (3%) and HIV/AIDS with 547 DALYs (2.1%). The potential proportion of our estimated TBE burden in this cluster amounts to 13.5%, demonstrating a relatively high burden in Slovenia. Thus, the present study can serve as an informative estimation of the TBE᾿s national burden and the importance of the TBE on population's health in Slovenia. TBE can be considered to have a high impact on public health and to present a challenge for more efficient health policies and actions to reduce TBE in Slovenia. Such action may lead to huge population health benefits on national scales.

Austria is the only European country where an extended vaccination campaign was launched in 1981. In Austria, vaccination is free of charge only for people with an occupational risk of TBE, while for the rest of the Austrian population a part of the costs are still covered by health insurance [9,80]. Vaccination coverage of persons receiving at least one dose of vaccine increased from 6% at the beginning of the programme to 88% in 2006, with 58% being regularly vaccinated within the recommended schedule [27]. This has led to drastic reductions in TBE in all age groups [27]. In the years between 2000 and 2006, about 2800 cases were prevented in Austria [27].

TBE is endemic in Slovenia, one of the countries with the highest incidence worldwide [30]. In Slovenia, vaccination is recommended for the general population from the age of one. TBE vaccine is free of charge only for those who are potentially exposed during fieldwork while the rest of the population has to pay for the vaccination [81,82]. Furthermore, because of limited public health resources in Slovenia, it was recommended [30] to provide only selective access to free of charge vaccination for specific age groups in specific regions. The findings of the present study may suggest the need to prioritize prevention of TBE disease further and reallocation of limited public health resources in Slovenia, for example, for an extended TBE vaccination for all groups, ages and regions, potentially free of charge or at a reduced price. It has been shown before that extended TBE vaccination can be beneficial from an economic perspective [80]. Extended vaccination may result in major health benefits to the population as a whole as well as cost savings to the health-care system.

## Conclusions

TBE presents a relatively high burden in Slovenia, expressed in DALYs both from the population and individual perspectives. Public-health impact may justify reallocation of scarce budgets to better control TBE. In particular, continued awareness raising and corresponding increased vaccination coverage is needed to reduce TBE and its consequences in Slovenia.
